# Chemical modification of the 6'-amino cyclopropyl of abacavir eliminates HLA-B*57:01-restricted CD8+ T-cell activation without loss of antiviral activity

**DOI:** 10.1186/2045-7022-4-S3-P40

**Published:** 2014-07-18

**Authors:** Mohammad Alhaidari, Emma Yang, Neil Berry, Andrew Owen, Steve Clarke, Paul O'Neill, Dean Naisbitt, Kevin Park

**Affiliations:** 1 University of Liverpool, MRC Centre for Drug Safety Science, UK; 2 GlaxoSmithKline, Drug Metabolism & Pharmacokinetics, UK

## Background

Abacavir, a nucleoside reverse transcriptase inhibitor, is used within drug combination therapy to treat the HIV-1 virus. It is a guanosine-analogue pro-drug, and once metabolised to carbovir, it acts as a DNA chain terminator. Exposure to abacavir is associated with a high frequency of CD8+ T-cell-mediated hypersensitivity reactions in individuals carrying the HLA risk allele B*57:01. To activate T-cells, abacavir interacts directly with endogenous HLA-B*57:01, altering the repertoire of peptides displayed on the cell surface. Docking studies show that the amino cyclopropyl group at the 6-position of abacavir assists in the arrangement and interaction of peptides with the MHC binding pocket. We hypothesized that chemical modification of abacavir at this position would provide a new series of analogues with potent antiviral activity that do not activate abacavir-specific CD8+ T-cells.

## Methods

Fifteen analogues were synthesized and their anti-viral activity measured. T cell responses to these analogues was then measured using CD8+ clones generated from HLA-B*57:01+ donors.

## Results

Several analogues, including the key closely related compounds, retained antiviral activity, but displayed highly divergent T-cell responses. Three HLA-B*57:01 positive blood donors were selected to generate abacavir-responsive T-cell clones. IFN- release was visualized using ELISpot to measure abacavir-specific T-cell responses and to determine which analogues activated the T-cells. All of the abacavir-responsive clones secreted IFN-, in a drug-concentration-dependent fashion (EC50 [50% of maximal IFN- spot forming units] less than 20M for all clones). A minimum of 5 clones were used to compare abacavir specific responses against the analogues. Abacavir and N-propyl abacavir were equally potent at activating clones. In contrast, two very closely related abacavir analogues were devoid of T-cell activity.

## Conclusion

Our findings show that it is possible to block the T-cell activity of abacavir while maintaining anti-viral activity by simple chemical modification.

